# A scenario-based approach to predict energy demand and carbon emission of electric vehicles on the electric grid

**DOI:** 10.1007/s11356-022-21214-w

**Published:** 2022-06-08

**Authors:** Wai Ming Cheung

**Affiliations:** Faculty of Engineering and Environment, Department of Mechanical and Construction Engineering, University of Northumbria, Newcastle Upon Tyne, NE1 8ST UK

**Keywords:** Electric vehicles, Electrical grid, Energy demand, CO_2_ emissions, Green vehicles

## Abstract

UK plans to ban the sale of new diesel and petrol cars by 2030 to be replaced by electric vehicles (EVs). The question is, will the UK’s electrical grid infrastructure ready for this change? This comparative study investigates the effect of UK green vehicles on the electrical grid and presents a new insight into improving their energy demand and carbon dioxide (CO_2_) emissions to the electrical grid. The results show that even when there is a very high level of market penetration of EVs, the overall effect on annual energy consumption may seem minimal. On the contrary, the effect that EVs may have on the electrical grid is dependent on the time-of-day EVs are being charged. Therefore, this study concludes that measures need to be put in place to control charging times of EVs and this would help restrict the total daily electricity and electrical energy demands. The introduction of EVs reduces the overall CO_2_ emissions mainly because a proportion of petrol and diesel cars are replaced by EVs. However, CO_2_ emissions can only reduce up to a certain level and this reduction of CO_2_ will have less effect due to an increasing number of EVs in the electrical grid. To reduce CO_2_ emissions further, the electricity that relies on high-carbon fossil fuels in the electrical grid should be set at the minimum level.

## Introduction

There are a variety of passenger electric vehicles (EVs) being developed by manufacturers which have a range of 350 miles before needing a charge (Park 2018). It is predicted that the market share of EVs in the future will be extensive, with 25% of all newly purchased vehicles being EVs from 5 million in 2018 increasing to over 40 million in 2030 globally (Global EV Outlook 2019). A substantial work has been carried out on EVs but there is minimal research focusing on the impacts of these vehicles on the UK electrical grid (Kapustin and Grushevenko 2020). These EVs require different amounts of energy to be charged from the electrical grid. Significant impacts are expected to occur on the power distribution grid due to the high energy required to power these EVs (Morrissey et al. 2016).

In addition to the impact of energy demand of EVs in an electrical grid, Köne and Büke (2010)’s finding suggests that 94% of the energy required to power these vehicles are carbon dioxide (CO_2_) intensive sources such as oil and coal. In 2007, road transport was responsible for approximately 17% of global CO_2_ emissions and is expected to consume around 44% of all energy consumed by 2050 (Paladugula et al. 2018). With increasing number of cars on the roads, improving their efficiency is crucial, and EVs are widely discussed as one of the main technologies to combat environmental impacts (Hawkins et al. 2012). UK plans to reduce emissions by at least 68% by 2030 compared to their 1990 levels (Department for Business, Energy & Industrial Strategy 2020). Furthermore, UK has also committed to achieve ‘net zero’ by 2050 (Bahaj et al. 2021). Therefore, reducing CO_2_ emissions in the transportation sector is globally important (Chen et al. 2020).

Petrol and diesel cars will gradually be phased out by 2030 in the UK (Sithole et al. 2016). To switch to EVs by 2030, this will introduce complexities into the electrical grid (Logan et al. 2020). Motoring experts warn that this demand for electricity will increase by 50% which will place unprecedented strain on the UK’s electrical grid (National Grid. 2017). One reason being there is no way of predicting when and where the vehicles will require energy. Additional battery load may occur at times when the electricity supply system is already heavily loaded (Qian et al. 2010). In this context, the impacts will be across the entire power system and hence, the impacts of EVs on the UK electrical grid need to be evaluated before they become heavily embedded into the transportation sector. This research therefore evaluates the pressures that EVs will place on the UK’s electrical grid in terms of energy demand and CO_2_ emissions.

## Literature review

This review highlights the key findings of energy demand and CO_2_ emissions on the electrical grid because of the development of EVs. A research gap has been identified in this review and this led to the proposed method that this work is addressed.

### Impacts of EVs on the electrical grid

There is a growing concern for both energy conservation and environmental protection means the development of EVs have been accelerated worldwide (Adnan et al. 2018). McCarthy et al. (2007) developed a simplified dispatch model to investigate impacts of integrating EVs into California’s energy system. The authors indicate that further research needs to be carried out on the effects of a large number of EVs connecting to an electrical grid around the same time. García-Villalobos et al. (2014) also stated that local network problems could be an issue depending on distribution network capacity and regional concentration of EVs. Shao et al. (2009) investigated the impacts of charging EVs on a typical distribution network in the USA. The investigation compared the results when EVs were charged at peak and off-peak times of a typical day. When EVs were charged at peak time, the grid’s transformers were overloaded and the efficiency of the transformers was reduced. The research suggests two strategies that could be implemented to prevent this overload: stagger charge and load control.

Perujo and Ciuffo (2009) evaluated the potential impacts to the electrical grid for the province of Milan. The key features they suggest investigating are the potential market penetration and the main technical features of the EV fleet. Hadley and Tsvetkova (2009) developed a scenario where the market share of EVs increased from 0% in 2010 to 25% in 2020. The model then sustained at 25% for the next decade. The report shows that the increasing market penetration of vehicles will raise additional strains on the electrical grid.

A similar study by Harris (2009) investigating the impact of the energy requirements of an increased number of EVs on the UK electrical grid in short and medium term. It is found that the electrical grid capacity should be adequate for a 10% market penetration of EVs. However, as EVs are still in early stages of production, it is hard to estimate future trends of this type of vehicles because market response and technological advances will affect EV development. A report by Shafiee et al. (2013) examined the impacts of changing levels of EVs on the electrical grid. The authors found that if 10% of the current fleet of cars in the USA turned electric, then the electrical load would increase by 31.35 GW. The research stresses the need to evaluate the effects of increasing the percentage of EVs on the road.

Based on the above review, a research gap on market penetration for EVs has been established as a topic of extensive research. To gain an idea of the impact of increasing numbers of EVs in the UK, this study considers three scenarios.

1. Slow progression of growth of EVs from 2014 to 2030. This scenario creates a situation where in 2030, EVs make up 10% of the car fleet in the UK.
2. Intermediate progression of growth of EVs from 2014 to 2030. This scenario creates a situation where in 2030, EVs make up 15% of the car fleet in the UK.
3. Fast progression of growth of EVs from 2014 to 2030. This scenario creates a situation where in 2030, EVs make up 25% of the car fleet in the UK.

### *CO*_*2*_* emissions on EVs*

CO_2_ is the most appropriate indicator to evaluate the environmental impacts of switching to EVs (Zhao et al. 2021). From an environmental perspective, the replacement of internal combustion engine vehicles with EVs may be beneficial for the climate because of the potential reduction of greenhouse gas (GHG) emissions (Thiel et al. 2010). One criticism of EVs is that they simply transfer CO_2_ emissions from the vehicles exhaust to power plants (Razeghi et al. 2011). Air emissions resulting from electricity production depend on the fuel mix and this differs by country and varies over time (Doucette and McCulloch. 2011). These differences in type, size, and location of emissions need to be taken into account to give an overall picture of the environmental impacts.

The effects that EVs may have on the electrical grid are covered by Kapustin and Grushevenko (2020). The article suggests that EV is one of the best methods to reduce current CO_2_ emission levels; however, there are some limiting factors, for example: the number of EVs in a region, supply and demand for EVs in that region, and electrical needs in an area.

Richardson (2013) finds that EVs reduce the total amount of CO_2_ emissions, even in electricity systems with a high fraction of fossil fuel generation. This is due to the high efficiency of an electric motor compared to an internal combustion engine. The author suggests further research is needed to reduce air pollutant emissions from electricity production. Electricity consumption does not emit CO_2_ at the point of use; however, GHG intensity (gCO_2_-eq/kWh) of electricity used to charge vehicles is a key parameter to estimate GHG impact (Constantine and Meisterling 2008).

This part of the literature review determines that further investigation of CO_2_ emissions due to increasing use of EVs is equally as important as the effect of energy demand on an electrical grid. The basis of this background review indirectly leads to the proposed scenario-based approach. The methodology is discussed in the following section.

## Methodology

To access the overall energy demand and the environmental impacts that EVs have on the UK electrical grid, the fundamental elements are:

(i) To investigate the energy demand of an increased number of EVs on the current electricity system.
(ii) To evaluate the environmental impacts, this research focuses on the CO_2_ emissions produced by the EVs’ energy demand on the electrical grid.
(iii) In this work, Plug-in Hybrid Electric Vehicles (PHEVs) and EVs are considered the same for the purposes of calculations. This research assumes that PHEVs will be used in the same way as EVs and will mostly be battery-operated, with inbuilt internal combustion units only providing insignificant fraction of energy.

To evaluate the impact of EVs, three factors that influence the scenario-based simulation are:

(i) Total predicted number of cars in the ‘car fleet’.
(ii) Energy required recharging the ‘car fleet’.
(iii) Composition of the ‘car fleet’.

To gain an idea of the demand that EVs will have on the electrical grid, this research investigates the yearly requirement of electricity that EVs will require at different market penetration levels. This scenario is based on all EVs will only charge when they needed. This simulation assumes that an EV daily mileage for commuting trips is within a 20 miles range (Graham-Rowe et al. 2012).

### Assessment of the impacts of EVs on energy demand

By looking at the daily energy demand in more details, it is possible to assess the impact that EVs will have on the electrical grid. Evaluations of the daily energy demand are outlined as follows:

- The number of daily charges is multiplied by the number of EVs for the total number of daily charges of the entire fleet.
- The amount of the total electrical energy required is simply multiplying the value in (a) by the battery capacity and the battery efficiency.
- The amount of electricity required to charge an EV is simply used the battery capacity and divided by battery recharging time with the battery efficiency.
- A yearly total of electricity required by the fleet can be calculated by multiplying the daily energy required by 365 days.

**Fig. 1:**
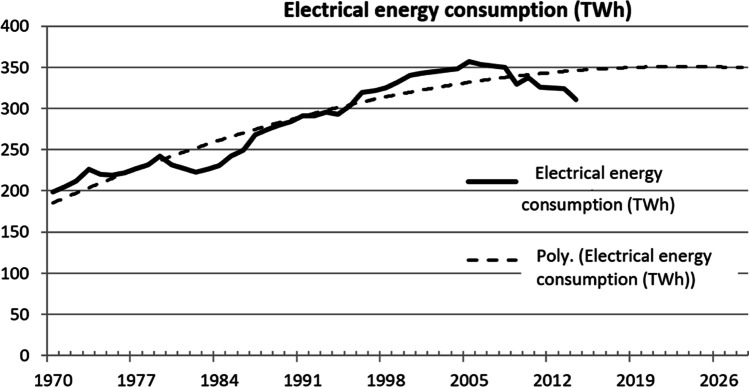
Actual and predicted electrical energy consumption UK 1970–2030 (Department of Energy and Climate Change 2020)

Figure 1 shows the predicted electrical energy consumption in the UK in 2030. This will act as a comparison to evaluate the impact on electricity demand due to EVs.

When assessing the potential impact of EVs on the electrical grid, two key elements must be considered.

- The core technical features for the available EVs need to be evaluated from short to medium term.
- The market penetration of the fleet of vehicles needs to be estimated in terms of the future trends.

The technical features of EVs will determine the potential market penetration. Further factors that need to consider are:

- The battery capacity of EVs, and
- The range or distance they can travel.
- Their energy consumption per unit of distance covered.

All these elements influence the type of commuter that will drive these vehicles. Currently, the range of EVs regards as being particularly small, some vehicles barely reaching 100 miles (Wu et al. 2015). This means that the vehicles need to be recharged more frequently and this process requires several hours depending on the energy available. Putrus et al. (2009) claim that slow charging from a single phase takes around 6 h. Since most of the people do not need to travel long distances, and therefore, this type of EVs is suitable for urban use. However, large urban cities are highly energy consuming areas, this means that these EVs may substantially suffer from electrical energy demand.

Table 1 shows a collection of EVs available in the UK. Table 2 indicates a summarised classification of EV fleets which have been clustered into specialised groups (small, medium, and large) depending on the capacity of the battery. EVs have different battery capacities means different energy are required by the electrical grid. Table 2 includes the expected recharging times for the EVs. Recharging time is very important as it helps to estimate the energy required by all the EVs. The recharging times vary for each EV. For this study, the investigation has considered an average recharging time of 6 h. This time is expected to decrease due to technological advancement.

**Table 1:** Available EVs in UK

Manufactures	Type of EVs	Battery capacity (kWh)	Average time to charge (hours)	Average distance travel (miles)	Consumption (kWh/100 Miles)
Nissan	LEAF	24	5	124	19.35
Mitsubishi	Outlander	12	5	32	35.29
BMW	i3	22	4	80	27.5
Renault	Zoe	22	6	149	14.77
Tesla	Model S	85	5	265	32.08
Kia	Soul	27	6	132	20.45
Volkswagen	E-UP	18.7	6	93	20.11
Ford	Focus-Electric	23	5	76	30.26
Audi	E-Tron	71	7	150	47.33

**Table 2:** Specialised groups of EVs

Segment	Battery capacity (kWh)	Time to charge (hours)		Distance travel (miles)	Consumption (kWh/100 Miles)
		Domestic	Fast		
Small	15	6	1	50	30
Medium	25	6	1	80	31.25
Large	35	6	1	110	31.81

The recharging power for each individual vehicle of an EV fleet is shown in Table 3. Domestic charging takes longer to recharge fully but requires less electrical energy from the grid. The fast-charging option demands a considerably larger amount of electricity from the electrical grid because it requires higher charging currents.

**Table 3:** Estimated electricity required to recharge an EV

EVs	Electricity required by grid (kW)	
	Domestic charging	Fast charging
Small	2.78	16.67
Medium	4.63	27.78
Large	6.48	38.89

Furthermore, the li-lon battery efficiency needs to be taken into account in determining how much electrical energy is required on the distribution grid. For this study, a battery efficiency of 90% is used (Richardson et al. 2011). The amount of electricity requires to recharge each EV can be determined by Eq. 1. The calculation assumes that each vehicle is fully charged once a day.

1 $$Electricity\;required=Battery\;capacity/(Battery\;recharging\;time\ast Battery\;efficiency)$$

The amount of electrical energy requires to recharge each EV can be determined by Eq. 2.

2 $$Electrical\;Energy\;required=Battery\;Capacity\ast Time\;to\;Charge\ast Battery\;efficiency$$

A huge uncertainty of this study is the potential market penetration of EVs in the UK. It is important to know the number of EVs in the future to gain an indication of their impact on the electrical grid. Many degrees of freedom of the uncertainty of the vehicle’s evolution could have a resultant impact of projecting the future progression of EVs. This study examines the market penetration of EVs from 2014 to 2030.

**Fig. 2:**
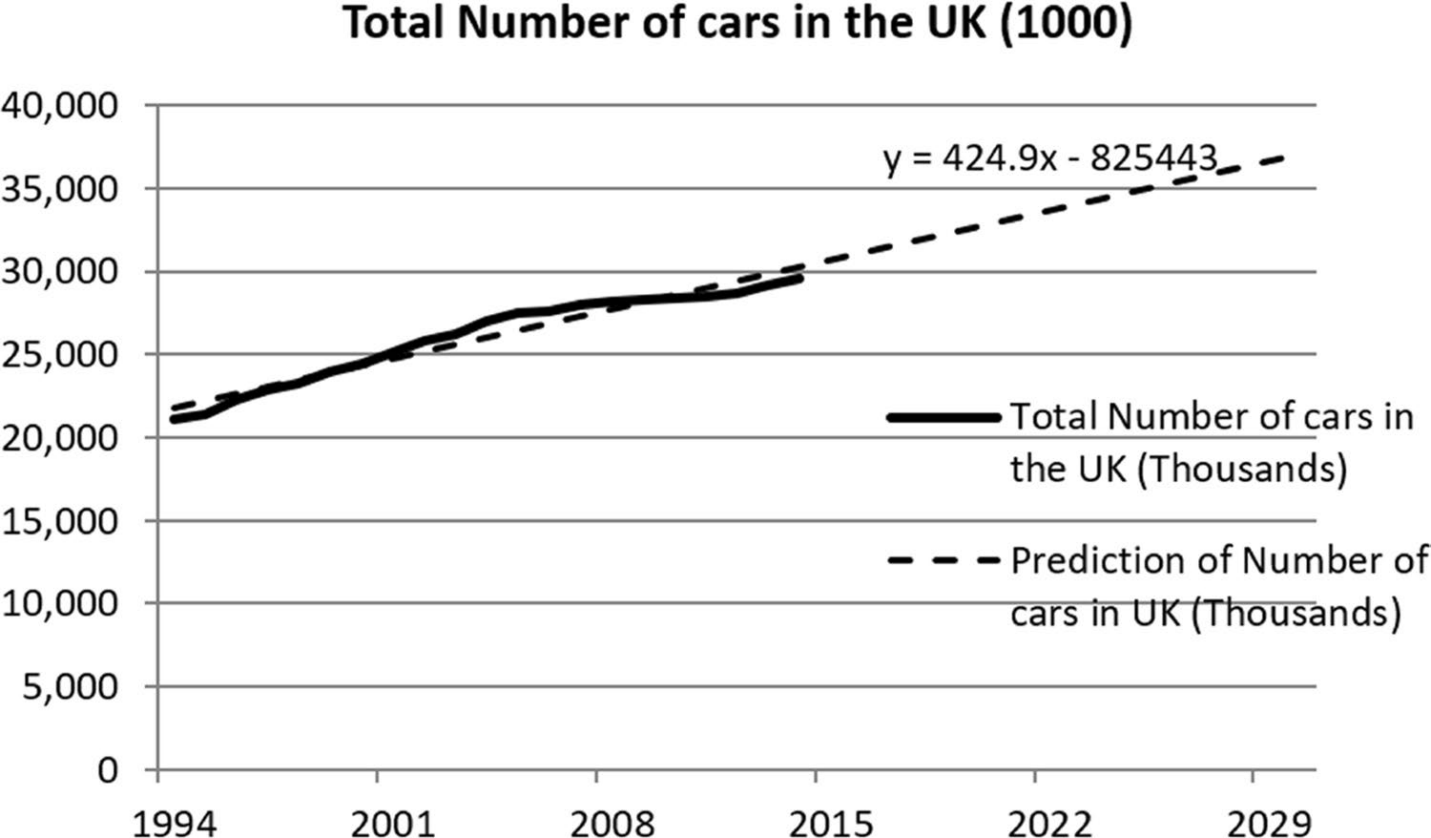
Total and predicted number of cars in the UK

This work investigates the impact on the electrical grid from the growth of EVs in the future. Transport Statistics Great Britain (2020) contains records of the number of registered cars in the UK dating back from 1970 to 2016. This information can be used to predict the number of EVs in the future as illustrated in Fig. 2. To estimate the market share of EVs in the UK, this can be obtained by using the predicted number of cars in the future with the three market penetration scenarios.

The composition of the vehicle fleet needs to be considered when investigating the total energy requirement of the EV fleet. By taking consideration of the new registrations by vehicle segment, it is possible to use the configuration specified in Table 4 to predict the amount of energy required by a future EV fleet. The percentage of electric car fleets of different segments is based on the UK’s new vehicle registrations in 2018 (Department of Transport 2019). To find out the number of cars of each vehicle segment, simply multiply the predicted number of cars of that year by the percentage as stated in Table 4.

**Table 4:** Composition of EVs

Vehicles by segment	Percentage (%) of electric car fleet
Small	35
Medium	44
Large	21

### *Assessment of CO*_*2*_* emissions on EVs*

EVs claim to have zero emissions but CO_2_ is still being emitted into the atmosphere as a result of electricity production. Therefore, to evaluate the impact of large deployment of EVs, the way in which electricity is produced must be considered. This part of the work focuses on evaluating the impact of EVs on the electrical grid in terms of CO_2_ emissions by considering different market penetrations. The factors that need to be taken account of are:

(i). Different market penetration levels of EVs will be considered ranging from 5 to 30%.
(ii). Different carbon emission factors for the ‘normal’ vehicle fleet will be considered.
(iii). To calculate the CO_2_ emissions produced by electricity generation, a carbon factor needs to be used. This factor remains constant throughout the model. It converts kWh of electricity to kgCO_2_ per mile travelled per car.

#### Energy mix in the UK

To find out the impact of EVs on CO_2_ emissions, information concerning the UK energy mix is necessary. The energy used to power the EVs is generated from a mix of production technologies which will produce a corresponding quantity of CO_2_ emissions. The energy mix contains information about what fuels are used to produce electricity and the percentage of usage.

#### Electricity CO_2_ factor

This work uses an electricity carbon factor to predict the CO_2_ emissions is based on the electricity used to power the EVs. The electricity carbon factor converts the electricity needed to power the car fleet in kWh to CO_2_ emissions (kgCO_2_). For this work, a carbon factor of 0.4585 is used (International Electricity Factors 2018). It is important to note that this carbon factor is an average and changes from year to year.

#### Range of miles

To access the impact of CO_2_ emissions, this study assumes that EVs will be used for commuting purposes with a driving range of 20 miles.

#### gCO_2_ per km

The CO_2_ emissions per kilometre of travel are converted to calculate the overall CO_2_ impact.

## Results

### Results of electricity and electrical energy demand by EVs

**Fig. 3:**
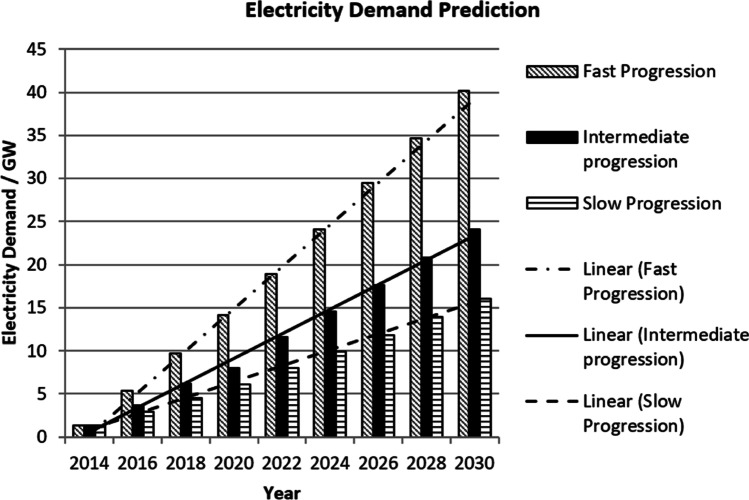
Results from the predictive model

Results of market penetrations of EVs over time and the electricity demand from EVs are shown in Fig. 3. In 2014, the percentage of EVs was 1% of the total cars in the UK, at this level, the EVs required between 1 and 1.5 GW of electricity to be fully charged. As shown in the results, it is feasible to gain a visual appearance of the impact the EVs have on the electrical grid.

The first scenario indicates a slow progression of increasing the number of EVs from 1% in 2014 to 10% in 2030. The results show that there is a positive correlation between the percentage of EVs in the UK and the amount of electricity needed. The peak electricity requires to charge the EVs is in the year 2030 due to having a larger percentage of vehicles being electric. When there is a 10% market penetration of EVs in 2030, the total electricity to power them is approximately 16 GW. This value is over 12 times the electricity needed to charge the EVs in 2014.

The second scenario accounts for an intermediate progression of cars in the UK from 2014 to 2030. By increasing the total number of EVs from 1% in 2014 to 15% in 2030, the amount of electricity needed to power the EVs peaks in 2030 at approximately 24 GW.

The third scenario predicts a fast progression of electric cars in the UK, starting at 1% in 2014 and increasing to 25% in 2030. The fast progression scenario requires the largest amount of energy to recharge the EVs. As indicated in the illustration, potentially 40 GW of electricity is required daily to charge EVs. This value is over thirty times larger than the electricity required for charging EVs in 2014.

Table 5 represents the results of the incidence of electric power demand of EV fleets from 1 to 30% in 2030. When the EV fleet is at the lowest value of 1%, the incidence on total electric energy consumption is the lowest. Hence, this only requires 0.26% of the yearly electric energy consumption. Even in the unlikely event when the EVs hold the largest share of 30% of total cars, the total electrical energy consumption requirement only reaches 7.86%.

**Table 5:** Incidence on total electrical energy consumption

Year 2030	Predicted total electrical energy consumption in the UK 2030 (350 TWh)						
Fleet share (%)	1	5	10	15	20	25	30
EV consumption (× 10^8^)	9.18	45.9	91.8	138	184	230	275
Incidence of EVs on total electric energy consumption (%)	0.26	1.31	2.62	3.93	5.24	6.55	7.86

### *Results of EVs CO*_*2*_* impact*

**Fig. 4:**
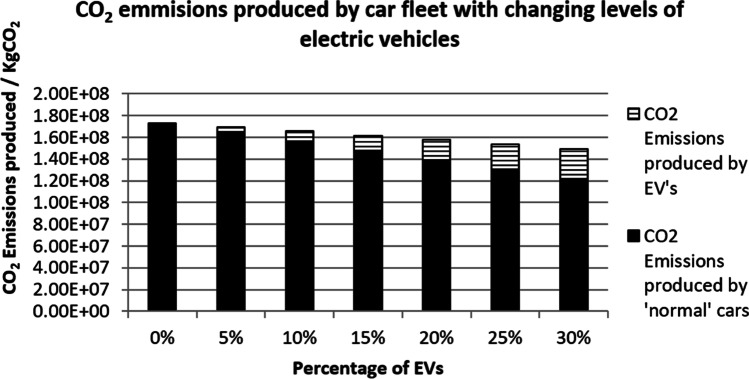
CO_2_ emissions (conventional cars 181 gCO_2_km)

This work has used three scenarios to predict the overall CO_2_ emissions on EVs. Figure 4 shows the result of the first scenario. The first scenario used an average car CO_2_ emissions data from the year 2000 where the total CO_2_ produced was 181 gCO_2_ per car per km (Transport Statistics Great Britain 2020). The total CO_2_ emissions produced by the ‘normal’ fleet and the EVs are combined together to gain a value for the total CO_2_ emissions. When there are no EVs present, the total CO_2_ emissions is much greater with a value of 1.73 × 10^8^ kgCO_2_. When the percentage of EVs increases, the total CO_2_ emissions decreases. The smallest amount of CO_2_ emissions is when the percentage of EVs is 30%, with a value of 1.49 × 10^8^ kgCO_2_. Furthermore, the total CO_2_ emissions fall below 13.48% from 0% of EVs to when there is a 30% of EVs.

**Fig. 5:**
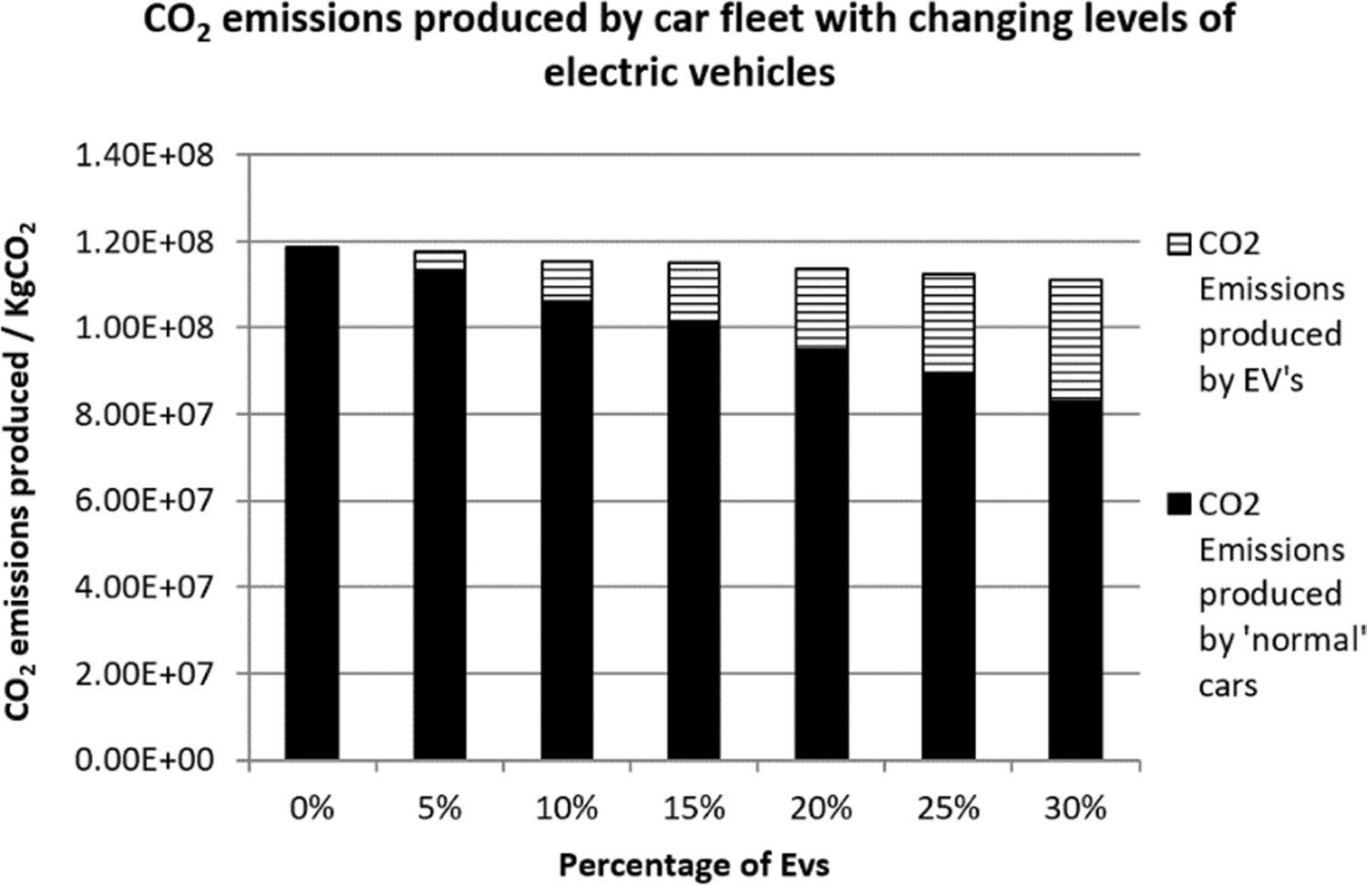
CO_2_ emissions (conventional cars 125 gCO_2_km)

Figure 5 shows the result of the second scenario. The second scenario used an average car CO_2_ emissions data from the year 2015, where the total CO_2_ emissions was 125 gCO_2_ per car per km. The overall CO_2_ emissions have reduced by a considerable amount of approximately 54 million kgCO_2_ which is due to a more efficient engine that produced less CO_2_ per km. However, the reduction of CO_2_ emissions is less at only 6.67% compared to 13.48%, despite the number of EVs have been increased.

**Fig. 6:**
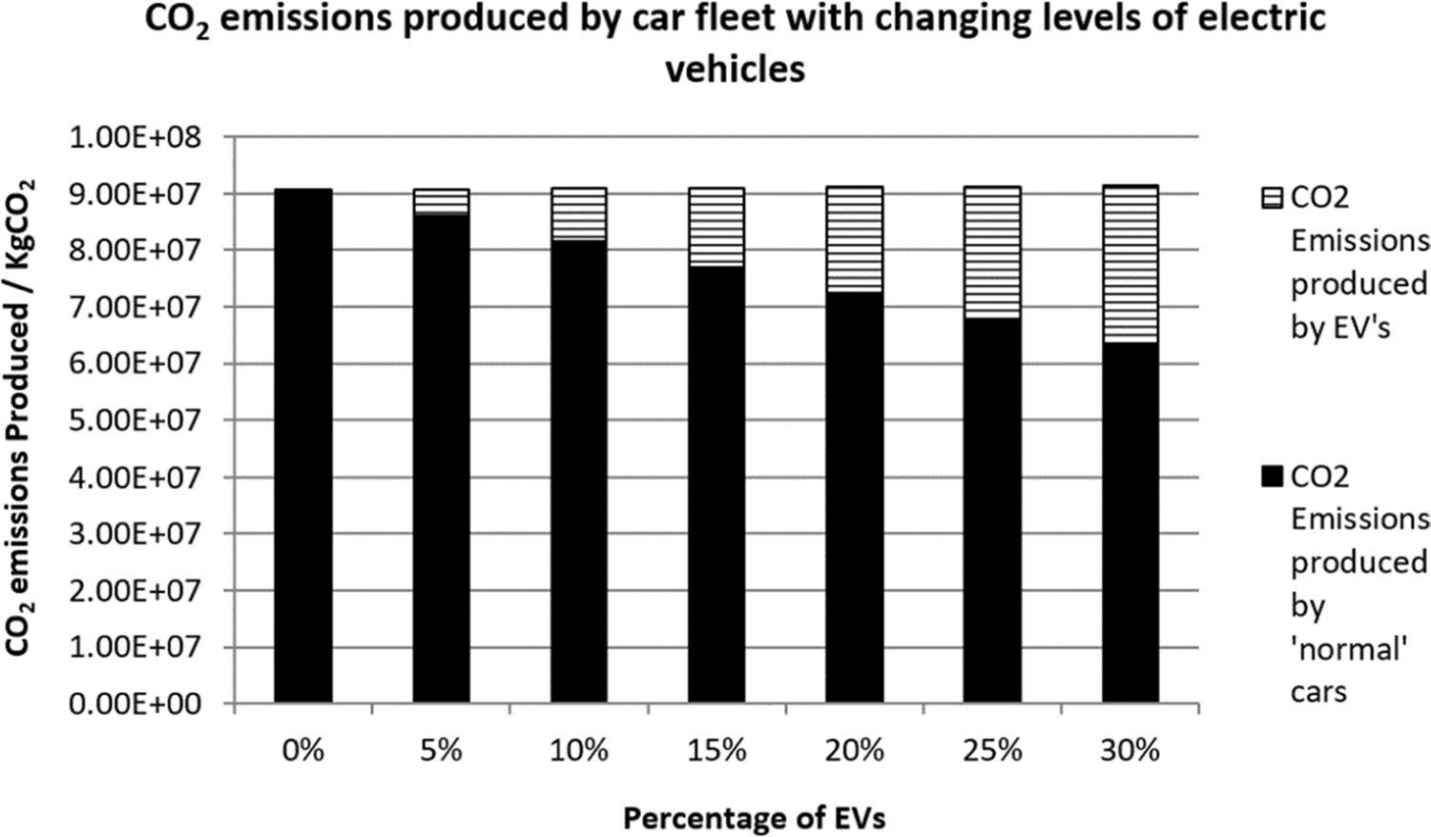
CO_2_ emissions (conventional cars 95 gCO_2_km)

Figure 6 shows the result of the third scenario. The third scenario used a target value of 95 gCO_2_km for the new car fleet in the year 2020 (The International Council on Clean Transportation 2016). This scenario predicts that the CO_2_ emissions produced by a vehicle from the ‘normal’ fleet is less than previous scenarios. This improved efficiency has produced 9 × 10^7^ kgCO_2_ even though when there are no EVs present in the fleet. However, the CO_2_ emissions have not reduced despite the percentage of EVs increased from 5 to 30%. The amount of CO_2_ emissions stays constant in this case. This suggests that the electricity carbon emission factor is restricting the CO_2_ emissions from improving.

## Discussions

**Fig. 7:**
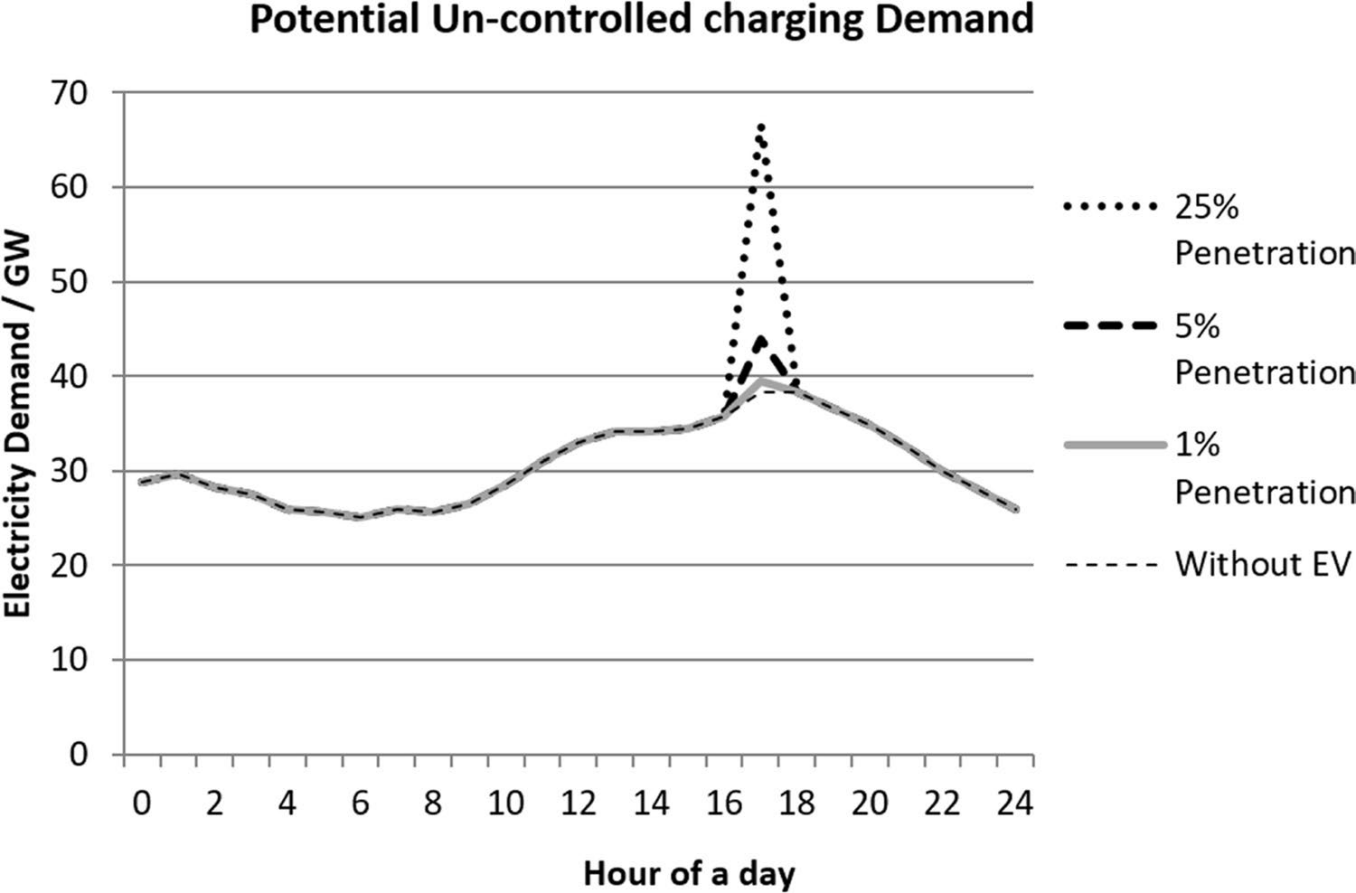
Impact on daily demand during uncontrolled charging

In Fig. 7, the baseline without EVs represents the daily peaks and troughs of average UK household electricity demand which usually occurs between 16:00 and 20:00 (Pimm et al. 2018). By taking consideration of this baseline, Fig. 7 shows the potential electricity demands could have had on the electrical grid if this uncontrolled charging of EVs took place. An increased number of EVs would only increase unprecedented pressure on the electrical grid. Even though it is unrealistic to assume that all the EVs are recharged in full capacity in daily basis, nevertheless, it shows the potential energy these EVs may require should uncontrolled charging takes place. When all the EVs were recharged daily at 25% penetration, they would require an additional 30 to 40 GW of electricity which would add a huge spike to the electricity demand.

For the electrical grid to cope with an increased number of EVs on the road, these EVs would need to be charged in a controllable environment. Co-ordinating charging can be done by a smart metering system. If the system was not adapted by this approach to charge at times when the demand is less, the electrical grid would need to be enforced. Upgrading the electrical grid would be the only option to cope with increased loads and voltage drops by heavy charging. Both options will add costs to the distribution system operators and eventually the customers.

From the results, it is clear to see that the introduction of EVs reduces the overall CO_2_ emissions mainly because a proportion of petrol and diesel cars are replaced by the EVs. Furthermore, the overall amount of CO_2_ produced by petrol and diesel cars will decrease over time as engines become more efficient. As shown in Fig. 6 where the normal car engines are predicted to have the most efficient engine, however, the CO_2_ emissions can only reduce up to a certain level and the percentage of reduction will have less effect due to an increasing number of EVs in the electrical grid. The overall reason for the percentage reduction of CO_2_ from an increased number of EVs is down to the fuels used to produce the electricity that power the EVs.

**Fig. 8:**
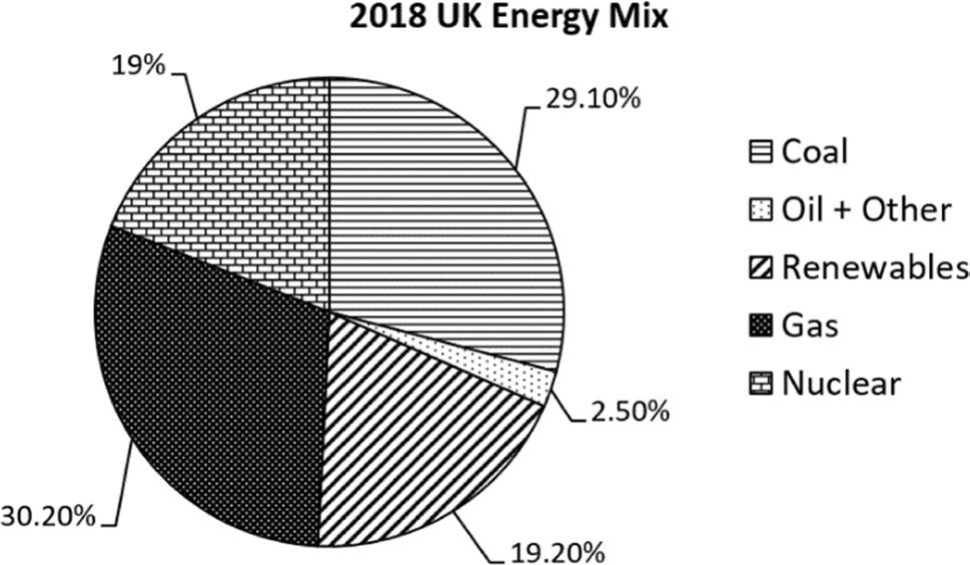
2018 UK energy mix

Figure 8 shows the UK energy mix from 2018 (Department for Business, Energy & Industrial Strategy 2018). This energy mix was made up from many fuel sources that produce a large amount of CO_2_, for example, 29.1% of electricity was produced by coal and 30.2% by gas. In 2018, the UK electricity produced from renewable sources accounted for a small proportion of the total electricity produced around 19.2%. The UK government aims to reduce GHG emissions by 68% of their 1990 levels by 2030. To achieve this, the electricity produced by low-carbon renewable resources in the UK must be increased and vice versa the electricity relies on high-carbon fossil fuels should be set at the minimum level. Consequently, the CO_2_ emissions produced by electricity generation in the future will be less and therefore EVs will be less carbon emissive than petrol and diesel vehicles. As the number of EVs increases and replaces many petrol and diesel cars, this means the environmental impact of the transport sector will improve.

## Conclusion and future work

The results show that even when there is a very high level of market penetration of EVs, the overall effect on annual energy consumption may seem minimal. Conversely, the effect that EVs may have on the electrical grid is dependent on the time-of-day EVs are being charged. When EVs occupy 25 to 30% of the total car fleet, they can have a substantial effect on the power demand. It can therefore be concluded that measures need to be put in place to control the daily charging time in EVs and this would help restrict the total daily power demand.

This work has also evaluated the potential CO_2_ reductions by increasing the number of EVs in the UK. The results show that the UK electrical grid would need to be upgraded as there are only positive results to be gained when EVs occupied a large market share. This research predicted that EVs do offer CO_2_ reduction potential on the electrical grid. However, it is essential to note that an extensive use of EVs may not actually contribute to the development of a sustainable transportation system. Even though EVs can reduce environmental stresses of road transportation, this is only one aspect of sustainable development. To progress towards a new paradigm of sustainable development, more effort is needed to pursue a collective transport system.

Future work to be undertaken needs to follow sustainable mobility. Firstly, there is limited peer-reviewed scientific literature specifically assessing techno-economics of a battery recharging infrastructure. Research needs to be conducted alongside industry and community stakeholders to support education and green vehicle readiness. Research needs to be actively involved in developing standards to support grid-friendly charging solutions to accommodate future trends of EVs.

## Data Availability

All related data and materials are within the manuscript.
